# A highly sensitive and specific method for the screening detection of genetically modified organisms based on digital PCR without pretreatment

**DOI:** 10.1038/srep12715

**Published:** 2015-08-04

**Authors:** Wei Fu, Pengyu Zhu, Chenguang Wang, Kunlun Huang, Zhixin Du, Wenying Tian, Qin Wang, Huiyu Wang, Wentao Xu, Shuifang Zhu

**Affiliations:** 1 The Institute of Plant Quarantine, Chinese Academy of Inspection and Quarantine, Beijing, 100029, China; 2 Laboratory of Food Safety, College of Food Science and Nutritional Engineering, China Agricultural University, Beijing 100083, China; 3 The Supervision, Inspection and Testing Center of Genetically Modified Organisms, Ministry of Agriculture, Beijing, 100083, China; 4 Guangxi Entry-Exit Inspection and Quarantine Bureau, Guangxi, 530028, China

## Abstract

Digital PCR has developed rapidly since it was first reported in the 1990s. It was recently reported that an improved method facilitated the detection of genetically modified organisms (GMOs). However, to use this improved method, the samples must be pretreated, which could introduce inaccuracy into the results. In our study, we explored a pretreatment-free digital PCR detection method for the screening for GMOs. We chose the CaMV35s promoter and the NOS terminator as the templates in our assay. To determine the specificity of our method, 9 events of GMOs were collected, including MON810, MON863, TC1507, MIR604, MIR162, GA21, T25, NK603 and Bt176. Moreover, the sensitivity, intra-laboratory and inter-laboratory reproducibility of our detection method were assessed. The results showed that the limit of detection of our method was 0.1%, which was lower than the labeling threshold level of the EU. The specificity and stability among the 9 events were consistent, respectively. The intra-laboratory and inter-laboratory reproducibility were both good. Finally, the perfect fitness for the detection of eight double-blind samples indicated the good practicability of our method. In conclusion, the method in our study would allow more sensitive, specific and stable screening detection of the GMO content of international trading products.

Within the last several decades, an increasing number of GM crops have been received the approval of cultivation and commercialization from different countries. In 2014, the number of hectares planted with GM crops reached 181 million, having risen 3.4% since 2013, more than 100-fold since 1996 and covering 10% of the total agricultural areas^1^. The public awareness of GMO crops has increased with the increased development of such crops. To protect the right to know of consumers for the products containing GMO content, many groups and countries have instituted labeling laws stating that the products must be labeled when they are above a certain threshold. Different countries have established different thresholds, such as 0.9% in the European Union (EU) and 5% in Japan, whereas the USA has voluntary labeling and China has “yes-or-no” labeling. Most of the labeling laws depend on the quantitative detection of GM crops, and thus the stricter laws must rely on more sensitive detection methods. Currently, the most commonly recommended method in the detection-standard documents of different groups and countries is the Taqman-based quantitative polymerase chain reaction (qPCR) method^2^,^3^,^4^,^5^. Many detection standards relied on qPCR results were established because of its relatively high levels of precision and accuracy^6^. However, this method has obvious drawbacks. The results of qPCR are analyzed according to the amplification curve^7^, which are easily affected by many factors, including the PCR inhibitor^8^, the experience of the technicians and the matrix effect^9^ of the PCR tubes. These drawbacks make qPCR unsuitable for screening the products with low-abundance GMO content.

Digital PCR^10^ is a recently developed quantitative detection method based on limiting dilution and statistical analysis based on Poisson Distribution. For this method, the original PCR mixture is partitioned into a series of reaction samples. Additionally, the number of templates in the diluted PCR samples follows Poisson distribution, such that most of the partitioned samples contain zero copies of the template and others contain one or more copies. After amplification, the partitioned samples containing one or more copies of the templates would exhibit fluorescence signals. By calculating the number of signal-exhibiting partitions and combining with Poisson distribution, the absolute copy number of the target templates in the PCR reaction volume is determined. By performing limiting dilution, the volume of each PCR reaction can be decreased to as little as nL or pL level, such that the “matrix effect” of the PCR reaction can be mostly avoided. dPCR has been widely used for the analysis of clinical samples, such as the detection of allelic discrimination^11^,^12^, the determination of single cell expression profiles^13^,^14^, the detection of single nucleotide polymorphisms^15^ (SNPs), and the detection of low-copy targets^16^,^17^,^18^,^19^,^20^. Additionally, studies have shown that dPCR is more resistant to PCR inhibitors compared with the real-time PCR^21^. Thus, dPCR may be less reliant on the purity of the DNA template. Therefore, the technique is better suited for international-trade applications.

However, the most important limitation to the widespread use of dPCR is the need to pretreat the genome samples. Previous studies showed that dPCR samples always required pretreatment, including restriction enzyme digestion^11^,^12^,^16^,^17^,^18^,^22^ and hot-process denaturation^23^. A recent study^23^ showed that the pretreated group allowed better separation of the target, the reference and the quencher cluster than did the non-pretreated group. Recycling procedure should always be followed by the pretreatments, while the column recycling kit is regarding as the most popular method as its easy operation and lower cost. However, this may cause the unbalance recycling for the gene fragments of different length as the different binding activity causing by the column of the kits. Thus, the pretreatments can lead to inaccurate detection results. Additionally, in the case of large-scale detection assays, pretreatments greatly increase the necessary time and labor.

Screening method based on the detection of screening elements of most of GMO events could be used to determine the GMO content within a short period. To promote the use of dPCR in routine testing, we evaluated a validated detection method based on screening pretreatment-free dPCR. Moreover, the intra-laboratory sensitivity, repeatability and specificity of our method were systematically evaluated. Finally, we sent our samples and our protocol to six different laboratories to confirm the inter-laboratory reproducibility of our method.

## Results

### Optimization of the experimental conditions

**Figure 1:**
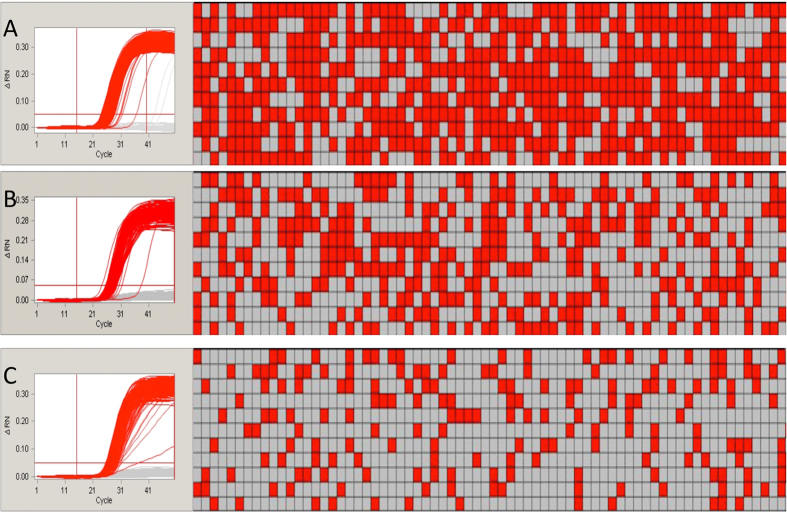
The amplification plots (left) and the panel hot map (right) of endogene and screening elements. The read wells of the each hot map mean the positive amplification, the grey one mean no amplification. (**A**,**B**,**C**) represented the group of ZssIIb gene, CaMV35s promoter and NOS terminator, respectively.

The thermal-cycling conditions, the concentration of probes and the mixing method were optimized in our assay. The final amplification plots and associated hot maps are shown in Fig. 1. Regarding the thermal-cycling conditions, studies^24^ have shown that the pre-denaturation duration may greatly affect the final results. Therefore, we chose a different pre-denaturation period for our PCR experiments: 120 s in our study and 300 s of officially recommendation. The amplification curves and hot maps showed that the shorter pre-denaturation time produced better final results. For the concentration of the probes, most of the validated real-time PCR detection methods used in China recommended a ratio of forward/reverse primers to probes of 2:2:1. To reduce the potential cost of our assay, we tested a forward/reverse primer to probes ratio of 9:9:2. The difference between the results of employing the two conditions was barely detectable. To improve the cost-efficiency of the method, we chose the ratio of 9:9:2 for further assays. For the aspect of mixing methods, two different methods were commonly utilized: pipetting or vortexing. We evaluated both of these methods and found that mixing by vortexing was more suitable for our experiments because pipetting can introduce inaccuracy due to DNA being left on the tips.

### Specificity and stability of the primers and probes

**Figure 2:**
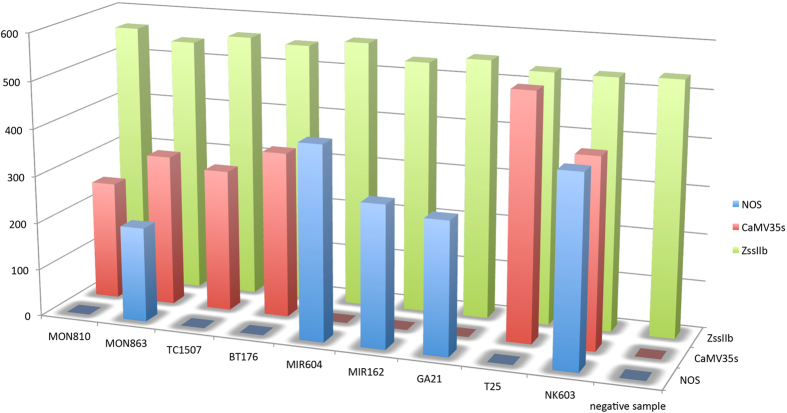
The specificity and stability test of our method.

In our study, specificity was theoretically defined as the unique amplification of the events that contained the correct screening elements. Stability was defined as the stable amplification of all of the events that contained the matched elements. The MON810, MON863, TC1507, BT176, MIR604, MIR162, GA21, T25 and NK603 events were used to test the specificity and stability of the primers and probes in our assay. The amplification results are shown in Fig. 2. The experimental amplification results all perfectly matched the theoretical results.

### Determination of sensitivity

In our study, the limit of detection (LOD) was defined as the lowest percentage of GMO content that could be reliably detected. To determine the sensitivity of our method, samples contained different percentages of Bt176 and MIR162 events were prepared. The sensitivity levels observed are shown in Table 1. The results showed that the positive wells coule be stably seen from the 0.1% group. Over the course of several repeated experiments, the 0.1% group reliably showed positivity. Thus, the LOD of this detection method was determined to be 0.1%.

### Validation of the repeatability

Intra-laboratory repeatability validation were performed by two different operators using two different chambers to determine the GMO content of the same sample on two different days. The validation results are shown in Table 1. The data in the sheets showed that all the different concentration of GMO samples appeared posititve results. This indicated that this dPCR detection assay provided reliably repeatable results for the sample at both high and low concentrations.

### Detection for the double-blind samples

To ascertain that this detection method was suitable for practical determinations of the GMO content, we conducted assays using eight double-blind samples that were mixed by coworkers in our laboratory. The GMO contents of the samples varied from 0.1% to 0.5%. The operators could not observe the theoretical data until the final practical results were available. The practical results and theoretical contents of the eight double-blind samples are shown in Table 2. The data showed that the practical results could be entirely fitted to the theoretical contents. Therefore, this dPCR detection method might be entirely suitable for the daily detection of GMO contents.

### Validation of reproducibility by inter-laboratory repeats

Reproducibility is one of the most important elements of concern for validated detection methods. To verify the reproducibility of the results obtained using the dPCR detection method, we sent our samples, primers and probes, as well as the experimental protocol, to six different laboratories to determine the level of inter-laboratory reproducibility. The results were returned with an official seal on the documents, meaning that the results had legal validity. The results verified by the different laboratories are shown in Table 3. We found that the results obtained in different laboratories were totally similar to the theoretical results. The similar results obtained in the different laboratories conclusively demonstrated the high level of reproducibility of our detection method.

### Validation of the repeatability by crossing-platform

**Figure 3:**
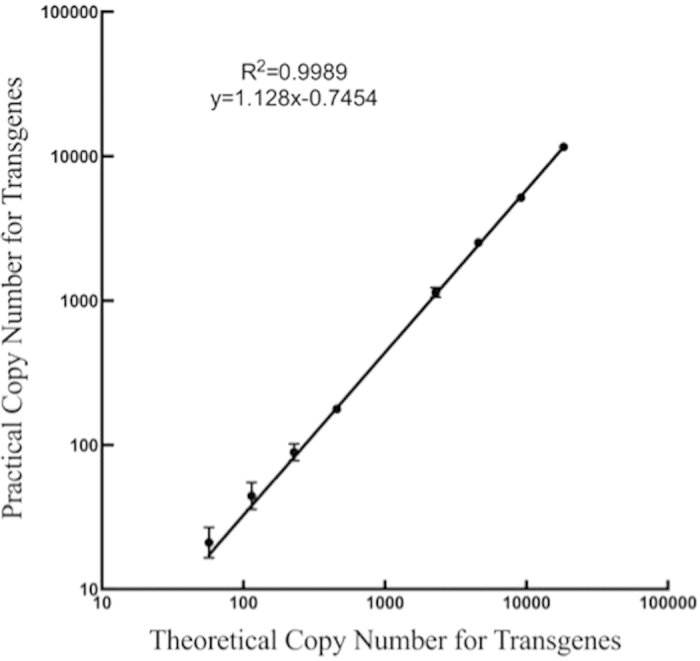
The linear curve between the theoretical copy number of transgenes and copy number of the screening elements.

The commercial platforms for conducting dPCR mainly involve two systems: the chamber-based digital PCR (cdPCR) and the droplet-based digital PCR (ddPCR) system. The cdPCR system is based on microfluidic chambers into which the reaction volume is partitioned, whereas the ddPCR system is based on droplets of water-in-oil emulsion. The different principles at play in these two systems may result in different detection results. We designed experiments to ascertain that our detection method was suited to these other dPCR platforms. The verification results are shown in Fig. 3. The results obtained using the ddPCR system indicated that the detection method we developed in this study had good inter-platform repeatability.

## Discussion

We developed an efficient GMO detection method based on dPCR that can be used to reliably detect a GMO content of 0.1% in genomic DNA samples that were not pre-treated. Having demonstrated the specificity, stability and repeatability of this method, as well as the reproducibility of its results at both the inter-laboratory and inter-platform levels, we have shown that our method is suitable for the detection of GMO content in international trading products.

**Figure 4:**
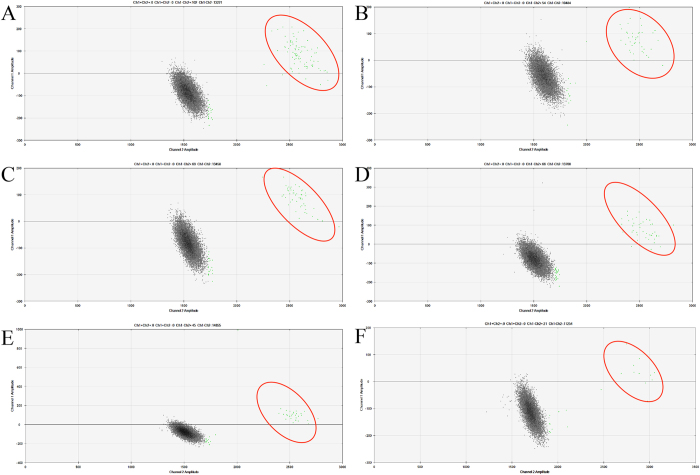
The amplification hot map of the verification of ddPCR with different GMO content. (**A**–**F**) represented the different GMO content of 1%, 0.5%, 0.4%, 0.3%, 0.2%, and 0.1%. The positive droplets were highlighted by the red cycles.

Previous studies showed that pretreating genomic DNA samples was essential to achieving reliable dPCR results. A recent study^8^ showed that pretreatments could be omitted from the ddPCR protocol; however, some researchers^17^ were concerned that without pretreatments, the stability of the method would be uncertain. For practical detection applications, including pretreatment steps makes the assays inconvenient and may introduce uncertainty into the final results. To comply with China’s labeling laws, routine qualitative detection methods must have good stability and specificity. The dPCR is the best choice for the detection of the low-abundance targets due to the partitioning of the initial PCR sample. Therefore, in this study, a stable pretreatment-free dPCR method for GMO screening was explored. Due to the production of random breaks in the genomic DNA during sample extraction, quantitative detection cannot be achieved by the screening elements. Thus, the target samples were serially diluted to evaluate the stability and reliability of our method. The linear curve of the theoretical copy number of transgenes compared with the practical number of copies of the screening elements per microliter is shown in Fig. 4. Based on the correlation coefficient of this curve, the linearity value of our method was 0.9989, showed that the good isolation of undigested genomic DNA by chambers even the GMO content is extremly low. This result suggested that the pretreatment-free dPCR detection method developed in this study had good sensitivity and linearity even when the GMO content was very low.

Two dPCR systems, the chamber-based dPCR (cdPCR) system and the droplet-based dPCR (ddPCR) system, were developed since the commercialization of the dPCR. Different results^11^,^16^,^17^,^24^ obtained using these two methods have been published, attributed to various factors, including the different principles of partitioning, the differential detection limits and dynamic ranges. The dPCR detection method that we developed should be suitable for both the cdPCR and ddPCR systems. Thus, the repeatability of our method was validated as being suitable for both of two dPCR systems. We used the 48.770 chamber manufactured by Fluidigm in our assay to generate 770 individual wells for each panel, compared with the 20,000 partitions generated in the Bio-Rad ddPCR system. Thus, the dynamic range of the ddPCR system may be greater than that of the chamber-based dPCR system. However, qualitative detection was achieved through the serial dilution of samples with various GMO contents by using our detection system. Accordingly, the dynamic range might not be the barrier for the comparison of two platforms. Moreover, the final results obtained proved our hypothesis that the cdPCR and ddPCR systems both could achieve a sensitivity level of 0.1%. The results indicated that the dPCR detection method that we developed achieved stable amplification using both two dPCR platforms.

We chose the CaMV35s promoter and the NOS terminator as the screening elements for our experiments. Previous studies^26^ showed that these two elements covered 65.7% and 53.49%, respectively, of all GMO events, and together, they covered 81.4% of these events, indicating that most GMO events could be detected using these two elements. Thus, we chose the CaMV35s promoter and the NOS terminator to complete our investigation. Using this approach, in the future, we will attempt to improve upon the method described here by adding more elements to cover a larger percentage of GMO events.

The dPCR screening method that we developed in this study allowed the detection of the samples with 9 GMO maize events, using one non-GMO maize sample as a negative control, simutanously. Moreover, the LOD of our method, which was 0.1% of GMO mass content, was lower than that of the GMO labeling limit of EU for 0.9% and satisfied all of the quantification limits for GMO labeling in other countries. Thus, the experimental results of this study demonstrated that our method was suited for the routine detection of GMO content in internationally traded products.

## Methods

### GMO samples

The non-GMO samples utilized in our study were stored in our the Supervision, Inspection and Testing Center of Genetically Modified Organisms of the Ministry of Agriculture. The various GMO samples were kindly provided by established companies, such as the MIR162, Bt176, MIR604, and 3272 event-containing samples that were provided by Syngenta Seeds Inc. (California, US); the MON810, MON863, GA21 and NK603 event-containing samples that were provided by the Monsanto Company (St. Louis, Missouri, US); the TC1507 event-containing sample that was provided by DOW AgroSciences LLC (Indianapolis, Indiana, US); and the T25 event-containing sample that was provided by Bayer CropScience (Leverkusen, North Rhine-Westphalia, Germany). The samples containing Bt176 and MIR162 events were used as the positive control samples for detection based on the CaMV35s promoter and the NOS terminator, respectively. The screening elements containing in each GMO event are shown in Table 4.

### Samples with different GMO contents

Before extracting the genomic DNA, the grains of the various GMO samples were ground using a Retsch^®^ MM430 mixer. The grinding procedure consisted of 10 cycles of agitation for 20 s and resting for 1 min cooling to room temperature. The cooling steps protected the genomic DNA from heat-damage. After the grain samples were ground, samples with different GMO contents were prepared. In this study, the relative mass content of each GMO event was set to 50%, 20%, 5%, 1%, 0.5%, 0.4%, 0.3%, 0.2% and 0.1%. The mixed samples were then placed in Dynamic CM-200 mixer and shaken overnight to achieve equal distribution.

### Extraction of genomic DNA

The genomic DNA was extracted using the DNeasy Plant Mini Kit (Qiagen, Germany) from 50 μg of powdered samples containing various GMO events. The extraction procedure was conducted according to the official guidelines. The isolated genomic DNA was diluted using 60 μL of ddH_2_O. The concentration and purity of each sample were determined using NanoDrop N2000 (Thermo Fisher Scientific Inc, Wilmington, US) according to the OD_260_ and OD_280_ values. The samples were diluted to a final concentration of 50 ng/μL for our study.

### Primers and probes

The primers and probes used in our assay are listed in Table 5. The TAMRA quencher that is generally used for real-time PCR was replaced by the Black Hole Quencher 1 (BHQ-1) to reduce the fluorescence baseline values. All of the primers and probes were synthesized by Invitrogen (Life Technologies, California, US).

## Digital PCR

### Chamber-based digital PCR

Chamber-based PCR (cdPCR) was conducted using a BioMark system (Fluidigm, South San Francisco) equipped with a 48.770 digital array (Fluidigm). The 48.770 array had 48 individual panels, each of which contained 770 individual partitions. A 4-μL aliquot of the initial PCR reaction volume was added to each panel to achieve a final volume of approximately 654.5 nL (0.85 nL × 770) for cdPCR reaction.

The protocol of cdPCR consisted of four steps, including priming, transferring, loading and amplifying. All of the experimental procedures followed the official guidelines. The final 4 μL reaction volume contained 1× Taqman Gene Expression Mix containing the positive fluorescence signal ROX (Life Technologies, California, US), 2× sample loading reagent (Fluidigm, South San Francisco), forward and reverse screening or endogenous primers at a final concentration of 225 nM and 1 μL of 50 ng/μL undigested DNA template, with ddH_2_O for the rest of 4 μL, the concentration of the probes was optimized in the later experiments. The predicted number of copies of the endogenous gene in each panel was 3000 based on the average theoretical mass of *Zea mays* genome being 2.73 pg^27^. Three parallel assays of each sample were conducted. The final results were analyzed based on the results in all three parallel assays.

### Droplet-based digital PCR

Droplet-based digital PCR was performed using Bio-Rad QX100 droplet system (Bio-Rad, Pleasanton, CA, USA). Many studies^11^,^17^ have shown that this droplet system provided good repeatability.

The ddPCR reaction volume included 10 μL of ddPCR Master Mix (Bio-Rad, Pleasanton, CA), 0.45 μL of the forward and reverse primers, 0.1 μL of each probe at the same concentration used for optimized cdPCR, and 5 μL of 50 ng/μL DNA template, with ddH_2_O added to bring the final volume to 20 μL.

The optimized cycling program used for both the cdPCR and ddPCR assays was as follows: 50 °C for 5 min, 95 °C for 5 min, and then 50 cycles of 95 °C for 1 min and 60 °C for 1 min, after which the fluorescence signals were collected.

### Data analysis

After the PCR assays were performed, the curves were automatically analyzed using Digital PCR Analysis Software (Fluidigm, South San Francisco). The final fluorescence threshold manually set to 0.03 and the wells with Ct values of 25–45 regarded as positive.

We estimated the absolute copy number per panel according to the number of positive wells and the total number of partitions. According to the Poisson distribution, the copy number of each panel was calculated using the following equations^27^:
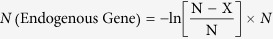

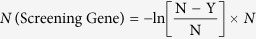
where the N (Endogenous Gene) value and N (Screening Gene) values were the estimated absolute copy number of the endogenous gene and the screening gene, respectively, and X and Y were the number of wells positive for the endogenous gene and the screening gene, respectively. N was the total number of separated partitions.

## Conclusion

We developed a validated qualitative screening detection based on pretreatment-free dPCR method in this study that enabled the detection of most of the commercially significant GMO events. This detection method achieved a lower LOD as well as a higher specificity, repeatability and reproducibility than that of the commonly utilized qPCR method, and it largely avoided false-positive detection results. Further studies using more screening elements would be conducted to cover a larger percentage of the commercially significant GMO events in more crops such as soybeans, maize, potatoes, rice and wheat.

## Additional Information

**How to cite this article**: Fu, W. *et al.* A highly sensitive and specific method for the screening detection of genetically modified organisms based on digital PCR without pretreatment. *Sci. Rep.* **5**, 12715; doi: 10.1038/srep12715 (2015).

## Figures and Tables

**Table 1:** The sensitivity and repeatability test.

**GMO content**^2^	**A**^1^								**B**								**The RSD of different operators for 35s**	**The RSD between different operators for NOS**
	**Three parallels of 35s**			**RSD**	**Three parallels of NOS**			**RSD**	**Three parallels of 35s**			**RSD**	**Three parallels of NOS**			**RSD**		
5%	142	136	138	2.20%	103	110	118	6.80%	145	142	149	2.42%	115	114	99	8.20%	3.02%	6.16%
1%	19	19	21	5.87%	15	14	17	9.96%	18	16	22	16.37%	14	12	12	9.12%	10.18%	12.37%
0.5%	6	4	6	21.65%	6	8	7	14.29%	5	5	7	20.38%	6	6	8	17.32%	17.41%	13.13%
0.4%	5	5	4	12.37%	5	4	4	13.32%	5	4	5	12.37%	4	6	4	24.74%	10.10%	16.97%
0.3%	4	2	3	33.33%	4	3	2	33.33%	5	2	5	43.30%	4	2	5	41.66%	35.95%	33.17%
0.2%	3	3	2	21.65%	4	2	2	43.30%	2	2	3	24.74%	3	5	2	45.83%	20.00%	38.49%
0.1%	1	1	2	43.30%	1	1	2	43.30%	1	3	2	50.00%	2	1	1	43.30%	44.72%	35.36%

^1^the A and B represents the different operators in our lab.

^2^the GMO content means the mass percentage of the GMO content.

**Table 2:** The theoretical contents and practical results for the double-blind samples.

**Group number**	**Theoretical GMO contents**	**Experimental P-35s results**	**Experimental T-NOS results**	**Fitness**
A	MON810 of 0.5%	Positive	Negative	100%
B	MON810 of 1%	Positive	Negative	100%
C	MON810 of 0.4% & MIR162 of 0.4%	Positive	Positive	100%
D	MIR604 of 0.2%	Positive	Positive	100%
E	MON810 of 0.3% & MIR604 of 0.2%	Positive	Positive	100%
F	NK603 of 0.4%	Positive	Positive	100%
G	Bt176 of 0.5%	Positive	Negative	100%
H	Negative maize sample	Negative	Negative	100%

**Table 3:**
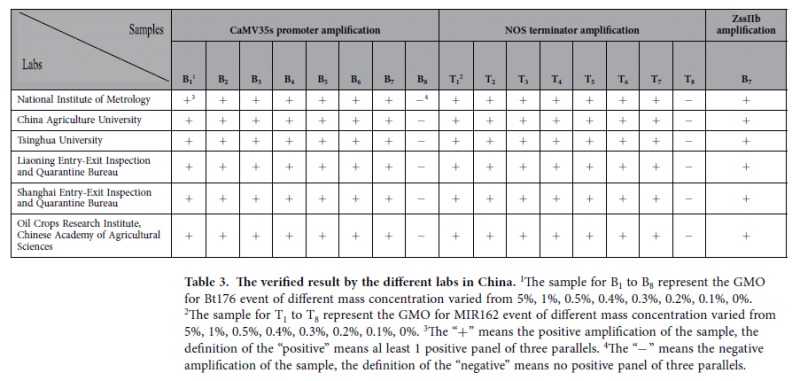
The verified result by the different labs in China.

^1^The sample for B_1_ to B_8_ represent the GMO for Bt176 event of different mass concentration varied from 5%, 1%, 0.5%, 0.4%, 0.3%, 0.2%, 0.1%, 0%.

^2^The sample for T_1_ to T_8_ represent the GMO for MIR162 event of different mass concentration varied from 5%, 1%, 0.5%, 0.4%, 0.3%, 0.2%, 0.1%, 0%.

^3^The “+” means the positive amplification of the sample, the definition of the “positive” means al least 1 positive panel of three parallels.

^4^The “−” means the negative amplification of the sample, the definition of the “negative” means no positive panel of three parallels.

**Table 4:** The screening elements contained in the events used in our study.

**GMO events**	**CaMV35s promoter**	**NOS terminator**
MON810	Positive	Negative
MON863	Positive	Positive
TC1507	Positive	Negative
MIR604	Negative	Positive
MIR162	Negative	Positive
GA21	Negative	Positive
T25	Positive	Negative
NK603	Positive	Positive
Bt176	Positive	Negative
Non-GMO	Negative	Negative

**Table 5:** The primers and probes used in this study.

**Target genes**	**Primers/probes name**	**Primers/probes sequence**	**Reference**
ZssIIb gene	ZssIIb-F	5′-CTCCCAATCCTTTGACATCTGC-3′	29
	ZssIIb-R	5′-TCGATTTCTCTCTTGGTGACAGG-3′	
	ZssIIb-P	5′-VIC-AGCAAAGTCAGAGCGCTGCAATGCA-BHQ1-3′	
CaMV35s promoter	P-35s-F	5′- ATTGATGTGATATCTCCACTGACGT-3′	30
	P-35s-R	5′- CCTCTCCAAATGAAATGAACTTCCT-3′	
	P-35s-P	5′-VIC- CCCACTATCCTTCGCAAGACCCTTCCT-BHQ1-3′	
NOS terminator	T-NOS-F	5′-ATCGTTCAAACATTTGGCA-3′	25
	T-NOS-R	5′-ATTGCGGGACTCTAATCATA-3′	
	T-NOS-P	5′-VIC-CATCGCAAGACCGGCAACAGG-BHQ1-3′	
